# Sirolimus-induced pulmonary toxicity without recurrence more than 8 years after everolimus replacement in a renal transplant patient with recurrent skin SCC: a case report

**DOI:** 10.1186/s12882-024-03661-4

**Published:** 2024-11-12

**Authors:** Golsa Ghasemi, Shahrzad Shahidi

**Affiliations:** 1 Isfahan Kidney Diseases Research Center, Isfahan University of Medical Sciences, Isfahan, Iran; 2 Isfahan Kidney Diseases Research Center, Khorshid Hospital, Isfahan University of Medical Sciences, Isfahan, Iran

**Keywords:** Kidney transplantation, mTOR inhibitors, Interstitial pneumonitis, Case report

## Abstract

**Background:**

Interstitial Pneumonitis (IP) is one of the pulmonary complications associated with mammalian Target of Rapamycin-Inhibitors (mTOR-Is). Sirolimus and everolimus belong to mTOR-Is. According to studies, IP is caused by both.

**Case presentation:**

This is a case report in a kidney transplant recipient. We want to present a case of IP after 50 months of sirolimus consumption. Sirolimus was discontinued, and cyclosporine was started. Thirty-seven months later, everolimus was prescribed as an alternative to cyclosporine due to the recurrence of skin Squamous Cell Carcinoma (SCC). Fortunately, no respiratory manifestations were seen after more than 8 years of everolimus consumption.

**Conclusions:**

In conclusion, in cases with sirolimus-induced IP, discontinuation of sirolimus and replacement with everolimus are recommended after resolving clinical symptoms and pulmonary lesions.

## Background

Renal transplantation is the definitive treatment of End Stage Renal Disease (ESRD). Taking immunosuppressive drugs is necessary to preserve the function of transplanted kidney [1]. Although immunosuppressive drugs play an important role for transplant patients, tumor growth is still a significant disadvantage for them [2, 3]. Calcineurin inhibitors (CNIs), as the main immunosuppressive agent, can develop post-transplantation cancer, and this effect could somehow limit their consumption in transplant patients [2]. Sirolimus and everolimus are immunosuppressive agents categorized as mTOR-Is [1].

In contrast with CNIs, mTOR-Is decrease the risk of cancer and increase the survival rate of patients in the future [3]. The mTOR-Is, like rapamycin, are popular due to their anticancer effect. Studies indicate cancers like renal cell carcinoma (RCC) and endocrine cancers that are angiogenic tumors can be treated with mTOR-Is. Many studies have shown that drugs similar to rapamycin, such as temsirolimus and everolimus, can be effective against cancers [4]. Therefore, mTOR-Is are less carcinogenic, similar to other drugs; some side effects are associated with them, e.g., impaired wound healing, metabolic abnormalities, proteinuria, and pulmonary complications [5–7]. IP is one of the pulmonary complications associated with mTOR-Is [6–8]. According to studies, the incidence rate of IP related to these drugs is 4-11% [6, 7]. The incidence of IP after sirolimus therapy in Iranian kidney transplanted patients was 9.7% [9]. This complication could appear early after the beginning of mTOR-Is or a few years later. Fatigue, fever, dyspnea, and cough are some non-specific manifestations of IP, which do not help the physician with clinical and immediate diagnosis. Chest X-Ray (CXR) is usually without abnormalities. However, ground glass opacities, bronchiolitis obliterans, and peripheral infiltrate patterns can be seen in Computed Tomography (CT) scans, especially in the lower lobes. As a result, diagnosis is difficult, and it must be made by excluding other diseases. Improving signs and symptoms after discontinuing the drug may be the only way to a confirmed diagnosis. The best way to treat IP is the discontinuation of mTOR-Is. According to some experts, high doses of corticosteroids may be recommended, although the exact dose of corticosteroids has yet to be mentioned in references [6, 10]. We want to present a case of IP due to sirolimus. Everolimus was prescribed as an alternative to cyclosporine after the recurrence of SCC. Fortunately, no respiratory manifestations were seen more than 8 years of everolimus consumption.

### Case presentation

**Fig. 1:**
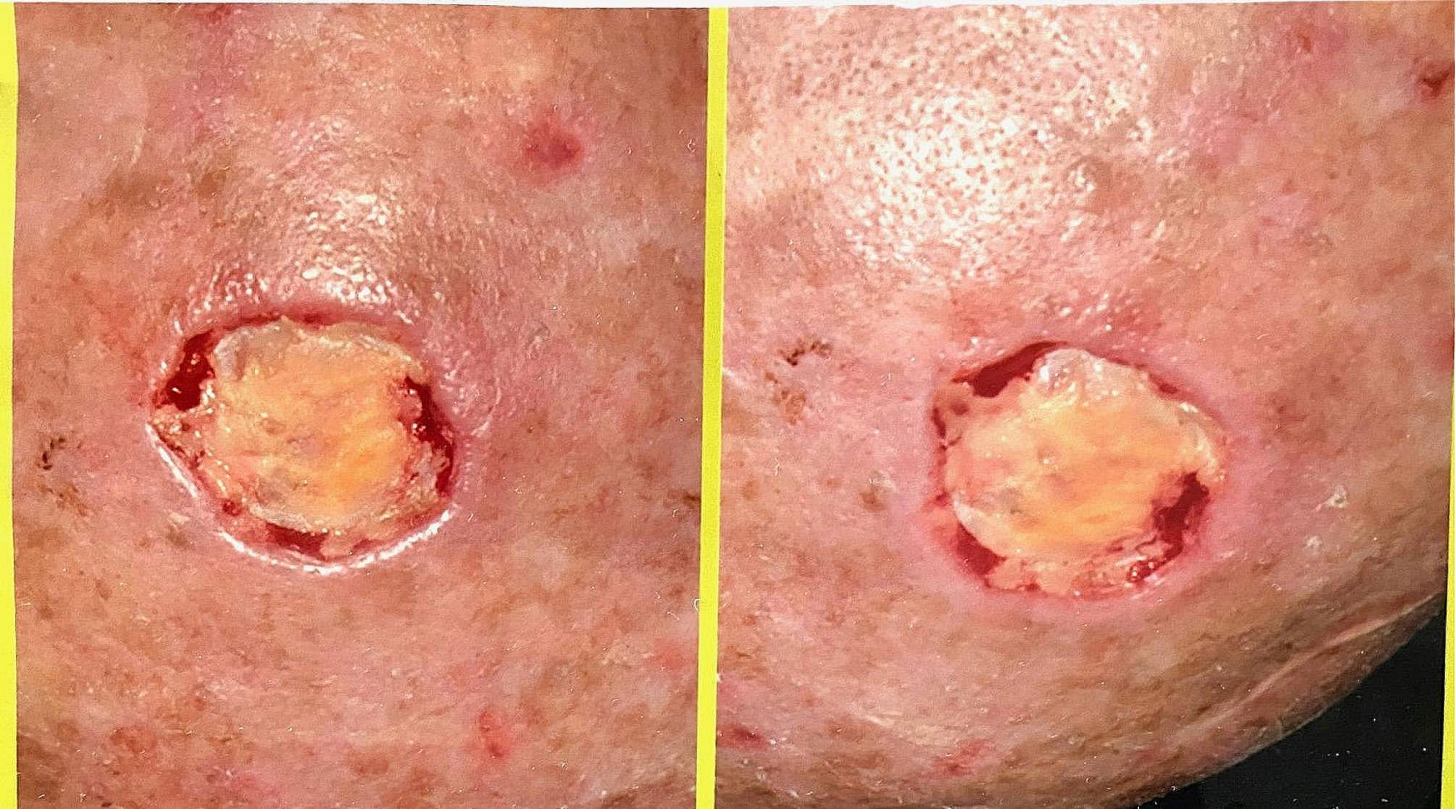
One of the SCC lesions on the patient’s head

**Fig. 2:**
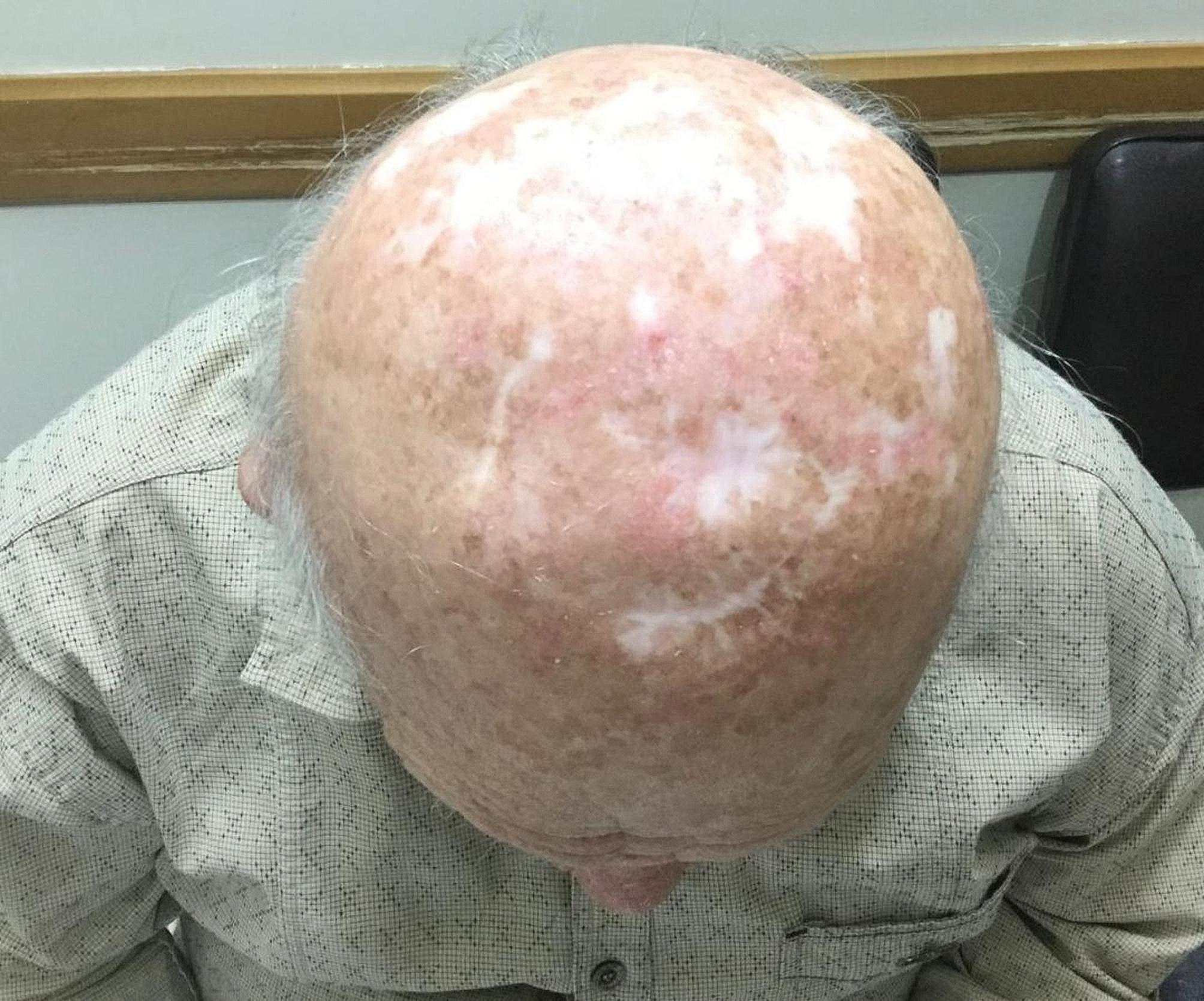
Scar of intensive SCC lesions on the patient’s head after healing

**Fig. 3:**
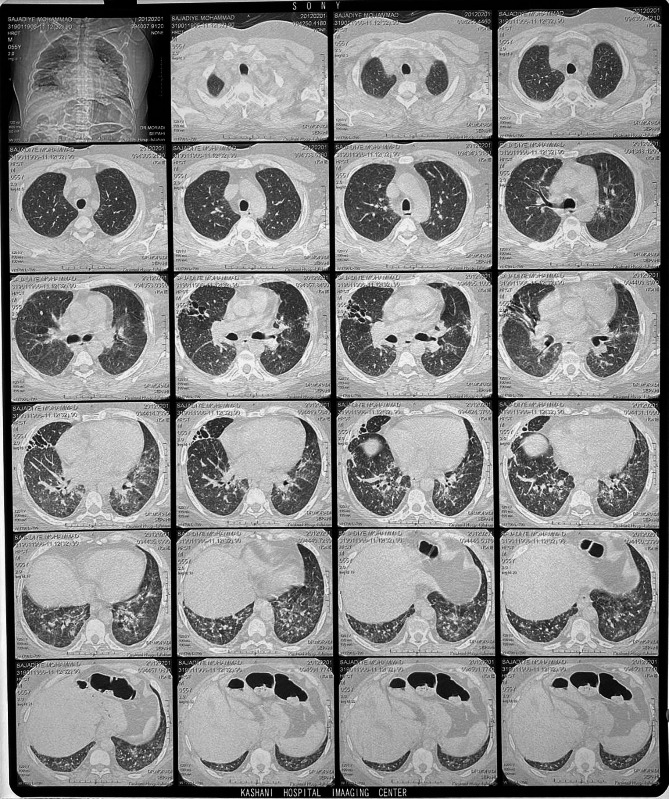
Extensive bilateral intralobular interstitial thickening and bronchiectasis is seen. Ground glass opacities are present in areas of reticulation. Subpleural sparing of the dorsal regions of the lower lobes is noted. Findings are consistent with fibrotic Non- Specific Interstitial Pneumonia (NSIPC)

**Fig. 4:**
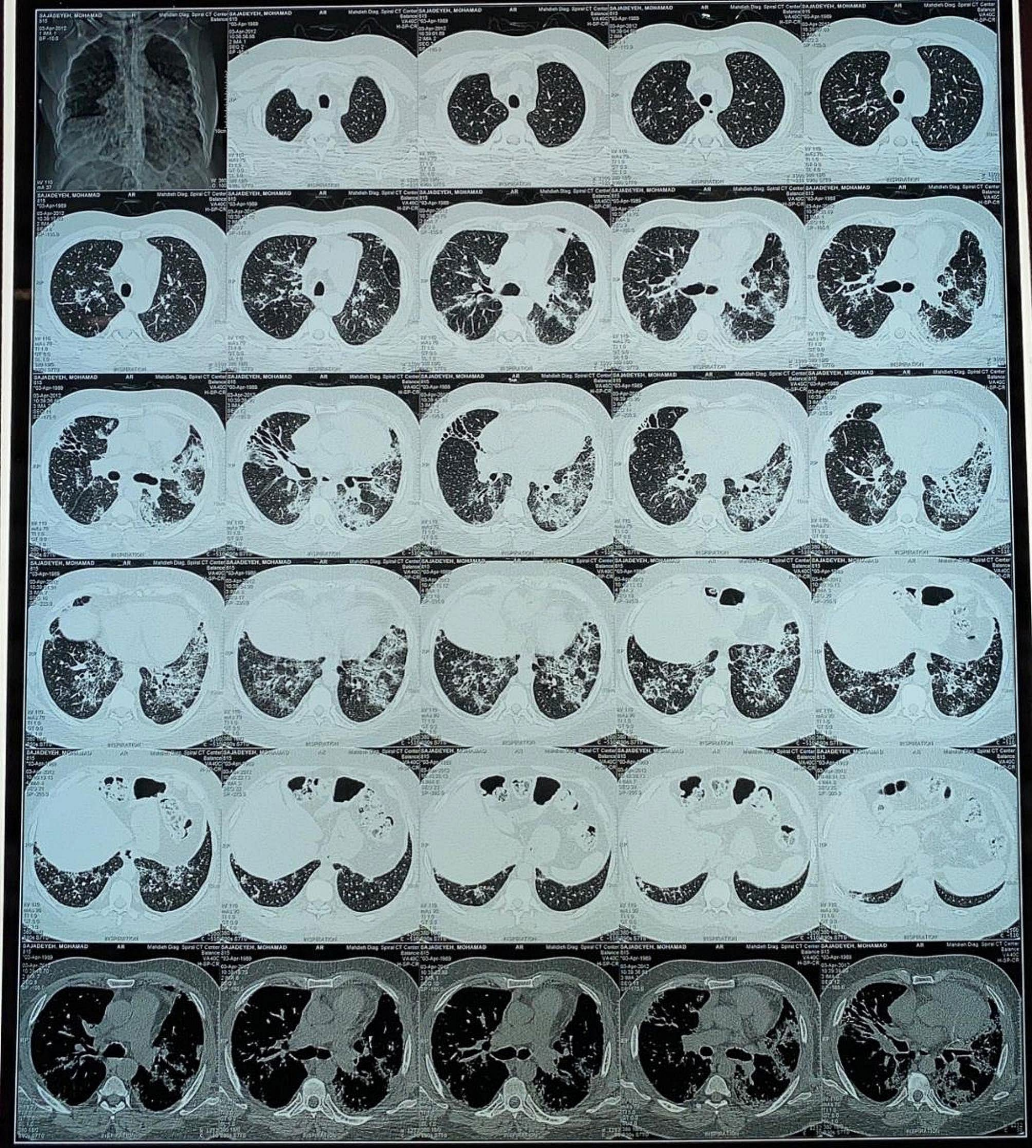
Some CT scan lesions disappeared after discontinuation of sirolimus

**Fig. 5:**
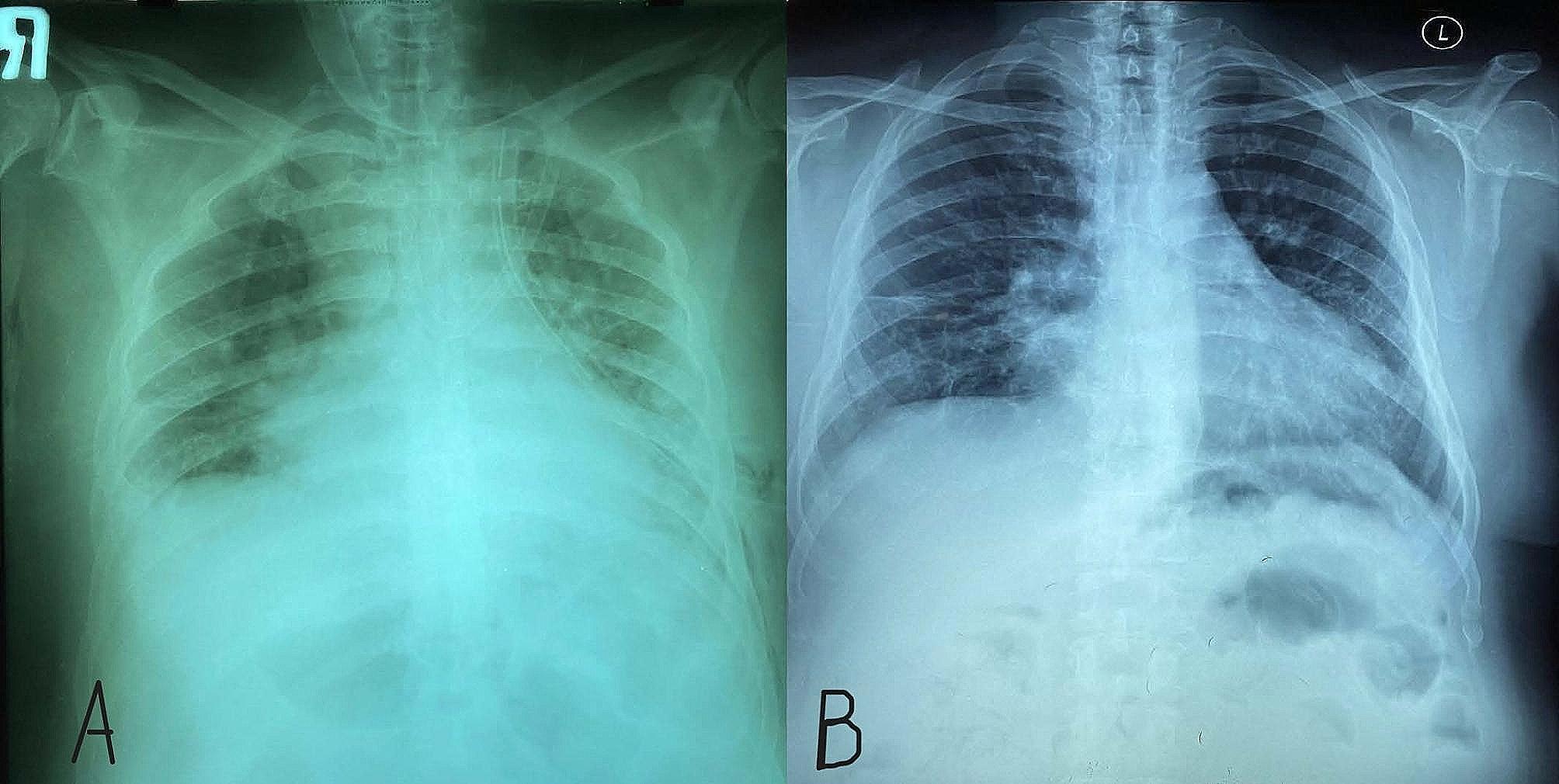
**A**. Onset of IP manifestations **B**. After discontinuation of sirolimus

A 47-year-old man with ESRD secondary to an unknown cause underwent a renal transplant on 7 May 2002 from a living-unrelated donor, a 20-year-old woman. His drug regimen included cyclosporine (Neoral^®^), prednisolone, and mycophenolate mofetil (Cellcept^®^). After five years of CNI therapy, multiple ulcerative lesions of SCC, which were diagnosed by biopsy, appeared on his head (Fig. 1). According to characteristics, SCC was high risk without lymph node invasion or distant metastasis. Serum creatinine (Cr), estimated-Glomerular Filtration Rate (eGFR), and cyclosporine level were 1.1 mg/dl, 78.8 ml/min/1.73m2, and 65 ng/ml (CKD-EPI 2021 update), respectively. After local treatment for SCC, sirolimus, as the alternative immunosuppressive, started. By the beginning of this drug, all head lesions had improved (Fig. 2). After 50 months of sirolimus therapy, non-specific manifestations appeared. Signs and symptoms included fever and dyspnea on exertion. The patient had a past medical history of gout, 8 years before the presentation of respiratory manifestations. Furthermore, he had a past medical history of lung disease. This problem has been started with pneumonia symptoms 2 years before renal transplantation. In the CXR, a homogenous lesion in the lower lobe of the right lung has been found. Chest CT scan considered two differential diagnosis for this lesion; the first one was lung tumor, and the second one was infection. The bronchoscopy and bronchoalveolar lavage (BAL) results were consistent with acute or chronic infection. Finally, a lung biopsy was performed, and the exact diagnosis was inflammatory pseudotumor. CXR manifestations were resolved 3 months later. In addition, New Onset of Diabetes after Transplantation (NODAT) 6 years after transplantation was diagnosed in the patient. Family history and psycho-social history were unremarkable. In the physical examination, a left axillary lymph node with a diameter of about 3 cm was detected. Lung auscultation was unremarkable. In the axillary lymph node biopsy, a non-specific reaction was reported. Chest CT scan suggested the possibility of malignancy or tuberculosis (TB) due to patchy bilateral infiltration (Fig. 3). A diagnostic bronchoscopy was performed. The result of the bronchoscopy was normal. Based on the pulmonary consultation, malignancy was a possible reason for these manifestations, and an open lung biopsy was suggested. For this reason, a transthoracic biopsy was performed. Tissue necrosis was the result of the transthoracic biopsy. Another differential diagnosis was infection (e.g., bacterial pneumonia, tuberculosis (TB), opportunistic bacteria, fungus, virus). A BAL sample was sent to the laboratory to be tested for TB, opportunistic bacteria, and fungus; after 2 months, the result was negative. Accessibility to polymerase chain reaction (PCR) for the diagnosis of respiratory viruses had some limitations. However, clinical features were inconsistent with respiratory viral infections. Lymphocytic alveolitis was the result of BAL cell types. Due to the possibility of drug toxicity, sirolimus was discontinued, and the corticosteroid dose was increased (50 mg/day). Constitutional symptoms, e.g., fever and dyspnea, have immediately improved. One month later, pulmonary manifestations resolved, and CT scan lesions disappeared (Fig. 4). Chest radiographs of the patient on the onset of respiratory manifestations (A) and one month after discontinuation of the sirolimus (B) are shown in Fig. 5. Over four months, the corticosteroid was tapered and reached a dose of 5 mg/day. Cyclosporine, as the main immunosuppressive, was again started. Unfortunately, after three months of cyclosporine therapy, intensive SCC lesions reappeared on his head. In the physical examination, the lesions were multiple crusted ulcers. Despite several times of surgical excision, skin lesions recurred. As a result, 37 months after the recurrence of SCC and no response to topical treatments (simple excision, curettage, and electrodesiccation), everolimus (Certican^®^) was started as an alternative to cyclosporine. During the last year of treatment, everolimus (Certican^®^) was changed to everolimus (Rolima^®^, NanoAlvand Inc.). Despite taking everolimus (1.5 mg/day) for more than 8 years, the patient shows no evidence in favor of IP caused by mTOR-Is. Last Cr and eGFR were 0.73 mg/dl and 99.1 ml/min/1.73m2 (CKD-EPI 2021 update), respectively. Everolimus level was 10.2 ng/ml in the last visit. For follow-up, intermittent private office visits and checking the trough level of everolimus are conducted every 3 three months. The interval of visits became longer after the stabilization of the patient’s situation and the trough level of the drug.

By the beginning of respiratory symptoms, the patient was nervous about the specific reason for his manifestations; however, after the diagnosis of IP and relief of his symptoms, he was delighted with the result. Although he was anxious about the best treatment for intensive SCC lesions on his head, he was pleased after the alteration of the drug and the disappearance of the lesions. The intervention adherence and tolerability were assessed by history taking, physical examination, and the trough level of the drug in each visit.

## Discussion and conclusions

MTOR-Is-induced IP is a problem after the beginning of treatment with mTOR-Is. Morbidity and mortality due to mTOR-Is-induced IP could be high; however, most of the time, it is mildly symptomatic or asymptomatic. While clinical manifestations in this type of IP are nonspecific, the diagnosis is difficult. A variety of differential diagnosis should be excluded before diagnosing mTOR-Is-induced IP. Malignancies, autoimmune diseases, and infections are examples of differential diagnosis for mTOR-Is-induced IP. BAL and lung biopsy were two diagnostic procedures to rule out other IP causes [11]. In this case, other IP causes had been ruled out by BAL and lung biopsy. After excluding other differential diagnosis, mTOR-Is-induced IP had been considered the most accurate diagnosis. Sirolimus is causing IP in solid organ transplant recipients and should be highly ranked between all causes of IP [12]. Studies showed that the interval between the onset of sirolimus therapy and sirolimus-induced pneumonitis is within 1 to 51 months. The reason for this wide range is not apparent yet [13]. Sirolimus-induced IP had been diagnosed after 50 months of sirolimus initiation in our patient. Contrary to the results of some studies that mTOR-Is-induced IP undoubtedly occurs with everolimus [14, 15], in this patient, despite a history of sirolimus-induced IP, pneumonitis did not develop after several years of everolimus use. The excretion of everolimus from the body is easier than sirolimus because everolimus is more hydrophilic. The possibility of hypersensitive reactions with everolimus is lower than with sirolimus [6]. Since 2010, everolimus has been used as an immunosuppressive agent after transplantation, and also this drug has been labeled as immunosuppressive therapy in advanced RCC and other malignances [10, 16]. Some risk factors may increase the incidence of mTOR-Is-induced IP, including past drug history of CNI therapy, renal dysfunction, smoking, and pre-existing pulmonary diseases [6, 15]. This patient’s risk factors of mTOR-Is-induced IP were a history of taking cyclosporine and pre-existing pulmonary disease.

It took about 3 months (85 days) to get the IP diagnosis, which is an important limitation of this study. Although an exact diagnosis took a long time, the diagnosis was precise, and the treatment had an appropriate result for the patient. Nonspecific signs and symptoms, unawareness of physicians about the disease, and lack of specific diagnostic tests to confirm the sirolimus-induced pneumonitis are some reasons for delayed diagnosis of sirolimus-induced pneumonitis [13]. Combining clinical data, radiographic findings, and bronchoscopy with BAL can help physicians accurately diagnose drug-induced interstitial lung disease [17]. Lymphocytic alveolitis is highly suggestive of sirolimus-induced pneumonitis. In this case, the high level of lymphocytes as a result of BAL showed a high probability of sirolimus-induced toxicity [13].

In cases where IP due to sirolimus appears, it can be discontinued and replaced with everolimus. This case report is one of the longest time reported on everolimus after mTOR-Is-induced IP without any pulmonary issues. The occurrence of IP with sirolimus does not necessarily mean that it will occur with everolimus, although both drugs have the same mechanisms. More extensive studies are necessary to confirm this recommendation.

## Data Availability

The datasets used and/or analyzed during the current study are available from the corresponding author upon reasonable request.

## Abbreviations

BAL: Bronchoalveolar Lavage
CKD-EPI: Chronic Kidney Disease-Epidemiology Collaboration
CNIs: Calcineurin inhibitors
Cr: Creatinine
CT: Computed Tomography
CXR: Chest X-Ray
eGFR: Estimated-Glomerular Filtration Rate
ESRD: End Stage Renal Disease
IP: Interstitial Pneumonitis
mTOR-Is: Mammalian Target of Rapamycin-Inhibitors
NSIPC: Non- Specific Interstitial Pneumonia
PCR: Polymerase Chain Reaction
RCC: Renal Cell Carcinoma
SCC: Squamous Cell Carcinoma
TB: Tuberculosis
